# Back to the Future—Past Learnings for Prospective Performance, Medicine and Health Research Recommendations in WOMEN’s Football

**DOI:** 10.1007/s40279-025-02250-1

**Published:** 2025-08-25

**Authors:** R. Lovell, K. Okholm Kryger

**Affiliations:** 1 Women’s Football Division, FIFA, Zurich, Switzerland; 2 Faculty of Science, Medicine and Health, University of Wollongong, Wollongong, NSW Australia; 3 Medical and Anti-Doping, Football Division, UEFA, Nyon, Switzerland; 4 Department of Sport and Exercise Sciences, Institute of Sport, Manchester Metropolitan University Institute of Sport, Manchester, UK; 5 Faculty of Sport, Technology and Health Sciences, St Mary’s University Twickenham, London, UK

## Abstract

The rapid professionalisation of women’s football has exposed significant gaps in biopsychosocial research that is essential to support player development, health, and career longevity. This current opinion examines historical deficiencies in research related to women’s football, and presents the WOMEN framework (World-wide representation, Open science, Methodology excellence, Evidence-based practice, Nurturing talent) to address these gaps and provide future research directions. Emphasis is placed on the necessity of diversity in research populations, adoption of open science practices and methodological rigor to produce impactful, generalisable findings. The framework also signifies the importance of research to support nurturing talent from youth to elite levels, considering the unique factors affecting female players. By learning from the past, and using available guidelines and resources, the existing knowledge gaps can be bridged to support evidence-based advancements in women's football, promoting both performance enhancement and player wellbeing across all stages of an athlete’s career.

## Introduction

The global rise of women’s football, with its increased participation and professionalisation, has highlighted the need for tailored, evidence-based research to address the specific health and performance challenges faced by women footballers. However, there remains a significant gap in research [1], particularly regarding critical areas such as female health, injury prevention and management, and optimising performance. This special edition has sought to address these pressing issues by providing evidence-based insights and recommendations that are essential for optimising performance and wellbeing throughout different stages of a player’s career, from youth development to motherhood and return-to-sport.

**Fig. 1:**
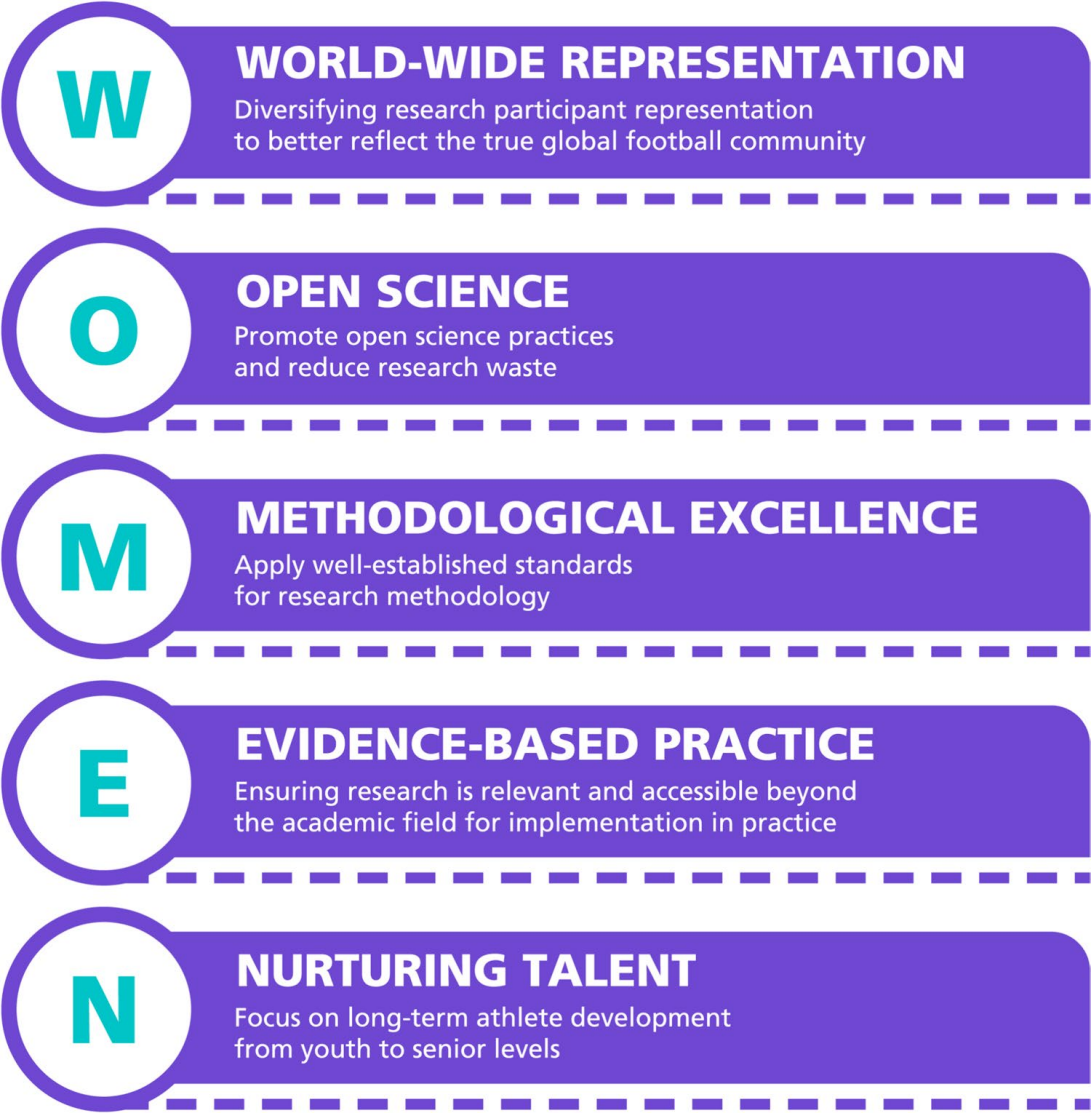
WOMEN football framework for advancing women’s football research

In this epilogue, we explore the notion of ‘back to the future’, drawing on lessons from past research practices to shape and improve the evidence-base to support women’s football professionalisation. By revisiting what has worked, and addressing what has been neglected, we can bridge the knowledge and application gap for evidence-based practice. To guide future research and practical implementation, this opinion piece introduces the WOMEN framework, a comprehensive approach that summarises key recommendations for advancing football research. The framework emphasises *World-wide representation, Open science, Methodology excellence, Evidence-based practice,* and *Nurturing talent* (Fig. 1). By fostering this framework and advancing research through rigorous, reproducible, multidisciplinary collaboration, the field of women’s football can bridge the existing data gaps and ensure that future practices are both scientifically sound and directly applicable to the diverse needs of female players. Furthermore, through prioritising research that directly addresses the unique biopsychosocial and developmental needs in women’s football, the findings can more effectively support long-term athlete health, performance and career longevity. By learning from the past and making strategic improvements, as a research community, we can move forward. This ‘back to the future’ approach serves as both a reflection and a roadmap for creating sustainable and impactful progress in women’s football research.

## W—World-Wide Representation

**Fig. 2:**
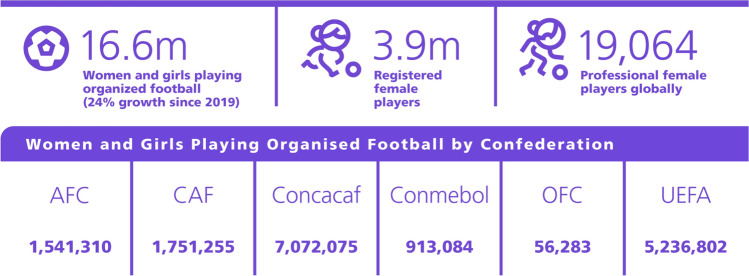
Participation figures from The FIFA Women’s Football Member Association Survey Report 2023 [2]

The current Fédération Internationale de Football Association (FIFA) campaign, ‘Football Unites the World’, reflects the sport’s unique capacity to bring people together across cultures, geographies and generations. Football is undeniably a global sport, and the global growth of women’s football across all age groups, FIFA football codes (association football, beach soccer and futsal) and competitive levels is evident. According to the 2023 FIFA Women’s Football Member Association Survey Report [2], there are now 16.6 million women and girls playing organised football—a 24% increase since 2019—including 19,064 professional players worldwide (Fig. 2). This surge in participation has also led to an increase in the number of coaches, referees, and medical and performance staff being involved in the women’s game. However, despite the participation growth, research on women’s football still lags in providing the necessary evidence-based practices [1]. Assuming that research findings from men’s football, or even from women’s football populations of different playing levels, football style (futsal, beach football, etc.), age groups and geographical regions can be universally applied to specific women’s football settings is an oversimplification and may lead to misinformation. Currently, most performance, health and medical research in women’s football is concentrated in Western European and North American football populations [3, 4]. Therefore, to truly support the global development of women’s football, greater diversity in research focus and translation is essential across different sub-populations. While initiatives such as the FIFA Football Nurse project [5] demonstrate promising steps toward inclusive, locally embedded research efforts, such programmes also face implementation challenges such as funding limitations, infrastructure gaps and the need for ongoing local capacity building. Equity, diversity and inclusion (EDI) statements and research grants are valuable (e.g. [6]), but insufficient on their own to generate sustained research outputs in underrepresented regions. Insights from broader global health and research domains suggest that long-term impact requires coordinated investment in local leadership, infrastructure and training; often supported through collaborative partnerships with established research institutions in high-capacity settings [7]. Football medicine and performance science can draw from these lessons to develop more durable, contextually relevant research ecosystems.

## O—Open Science

Although some progress has been made since the 2021 review [1], significant gaps in women’s football research persist. This presents a chance to learn from past shortcomings, reduce research waste and to implement more effective research strategies. By embracing transparency in the research process, engaging stakeholders in the determination of research priorities and fostering collaborative approaches we can enhance the quality and impact of research, ensuring its direct translation to football policymakers, support teams and players.

Open science promotes transparency through pre-registration of study designs. This approach mitigates issues such as selective reporting and hypothesising after results are known, which are often driven by pressure to produce novel findings and can lead to distorted scientific records and publication bias [8]. Open data platforms also promote the sharing of raw data and analysis code to foster replication and further interrogation (e.g. Dryad, Open Science Framework). Despite limited current adoption in sports medicine [9], researchers in women’s football performance and health can lead a cultural shift towards open science, where study registration, protocol availability, data sharing, and code availability become standard practices. By adhering to these practices, research gains credibility, facilitating confident implementation of findings in real-world settings.

Many essential research questions in women’s football require the need for robust data sharing and collaboration. Addressing research questions in female health and performance needs to reflect the natural degree of within- and between-player variability in physiology such as symptomology and hormonal systemic effects owing to reproductive status [10, 11], and often requires longitudinal data in volumes that may exceed the capacity of individual researchers or single clubs. Pooling data from multiple sources can enhance reproducibility and support more robust, generalisable findings, provided that consistent data collection protocols and tools are used across settings. Whilst the competitive nature of football provides some barriers to data sharing [12], such challenges can be overcome with robust ethical and data safeguarding measures. In addition, citizen science, which involves the participation of non-researchers such as players, coaches and community members in the collection and sharing of data to support research, may present a promising avenue for mitigating the impact of underpowered studies. By contributing valuable data on performance, injuries and participation trends, these stakeholders can help expand research reach and relevance. Creating accessible platforms for data transparency, providing training, establishing clear guidelines for data quality and privacy, and fostering partnerships with clubs and community organisations can ensure responsible and effective participation. This collaborative effort not only enables large-scale, cost-effective data collection but also strengthens the connection between research and the football community.

To further support the growth and professionalisation of women’s football, research prioritisation exercises that incorporate stakeholder engagement could be adopted in the pursuit of inclusive and sustainable research initiatives (e.g. [13–15]). The gender data gap provides an opportunity to conduct such exercises by identifying and focusing on the most critical research questions, ensuring resources are directed towards areas with the greatest potential impact [16, 17]. Engaging stakeholders—including players, coaches, administrators, medical professionals and policymakers—ensures that research agendas align with the needs and priorities of those who will apply the findings. Furthermore, the application of implementation science can enhance research translation by studying how best to integrate evidence-based findings into everyday practice. By systematically addressing barriers to adoption and ensuring effective dissemination, implementation science helps to bridge the gap between research and practice [18]. Collectively, these inclusive and methodical approaches not only enhance the relevance and applicability of research but also facilitate its effective translation into policy development and practical application, ensuring that advancements benefit all stakeholders in women’s football.

## M—Methodology Excellence

As researchers today, we are fortunate to have access to extensive guidance on maintaining methodological rigour through established guidelines and tools, which help optimise study design, presentation and methodological quality whilst aiming to minimise our research bias. Therefore, this section does not intend to propose novel methodological approaches for women’s football research. Rather, it underscores the importance of effectively applying these well-established tools to ensure rigorous methodology. Doing so will not only enhance data integrity and improve publication success but also facilitate the meaningful translation of research findings into practice.

In the high-stakes environment of elite women’s football, traditional methodologies such as randomised controlled trials, while considered the ‘gold standard’ in clinical research, are often impractical. The small sample sizes, competitive pressures and potential ethical concerns limit the feasibility of randomised controlled trials in sports settings [9]. Therefore, alternative methodologies such as pragmatic trials, *N*-of-1 designs, and natural experiments offer more flexible and effective solutions [9]. These approaches maintain methodological rigor while being more suited to the unique demands of football medicine and performance environments.

### Rigorous Research Design

Research rigor can be further enhanced through two key pre-study practices: employing ‘red teams’ and submitting protocols for peer review. Red teams, comprised of independent evaluators, critically assess the research design, methodology and potential biases, identifying weaknesses before study commencement [19]. Simultaneously, pre-study peer review of protocols enables early detection and correction of design flaws. These complementary approaches ensure methodological soundness and defensibility from the outset, strengthening the overall robustness of the research. The use of registered reports, where the study design and analysis plans are reviewed and accepted for publication before data collection, have also been introduced by journals in the field [20, 21]. Registered reports reduce the risk of publication bias and reinforce the distinction between exploratory and confirmatory research by committing researchers to their pre-registered hypotheses and methods. This approach ensures that negative or null results still contribute to a comprehensive understanding of female athlete health and performance. Despite these benefits, there has been limited uptake of registered reports in the sports science and medicine field. The additional time and effort required for registered reports and protocol submissions may necessitate more funding and resources, which are not always available. To address these, research stakeholders and governing agencies should encourage specific funding streams to support adoption within the research community.

### Reporting of Research Results

Research results should aim to be clear and transparent, making interpretation and critical assessment easier for readers to understand the findings. This can be achieved by following recognised guidelines, such as COSMOS-E [22], PERSiST [23] or PRISMA [24] for systematic reviews; STROBE [25] for observational studies; or TIDieR [26] and CERT [27] for randomised and non-randomised control trials. These tools help ensure consistency and completeness in reporting. It is also essential to ensure that research consumers are not left to self-interpret key terminology. In many cases, consensus statements are available to guide the consistent use of terminology, helping readers clearly understand key concepts whilst ensuring comparability across studies. For example, the football-specific extension of the IOC consensus statement for recording and reporting injury and illness data [28], and the female athlete health domain supplement for women’s football can offer valuable direction [29]. Similarly, systems such as the Football Injury Inciting Circumstances Classification [30] are useful for ensuring clarity. Where no standard terminology is available, or alignment with existing terms is challenging, offering clear definitions can enhance understanding and avoid misinterpretation.

Future research endeavours to advance women’s football health and performance may benefit from multi- and transdisciplinary collaborations that integrate diverse perspectives, methodologies and expertise from outside traditional football domains. Multi-disciplinary teams typically involve experts from various fields—such as sports medicine, psychology, physiology, nutrition, biomechanics, statistics and data science—who work together to address complex research questions. Transdisciplinary research, by contrast, transcends disciplinary boundaries, fostering the co-creation of novel frameworks and methodologies. This approach is particularly valuable in women's football research, where the intricate interplay of biological, psychological and social factors demands innovative investigative methods. Transdisciplinary teams integrate academic expertise with insights from non-academic stakeholders, including coaches, medical practitioners and athletes. Recent examples include the development of return-to-play protocols for postpartum athletes [31], combining clinical, performance and psychosocial expertise, and the FIFA concussion education initiative, which involved collaboration between researchers, medical staff and communication professionals [32, 33]. By grounding investigations in the practical context of football, these methods yield more holistic solutions for enhancing athlete health and performance [34]. Moreover, transdisciplinary research facilitates knowledge synthesis, effectively addressing the limitations inherent in traditional reductionist approaches often employed in performance studies [34].

Finally, we wish to emphasise that ensuring methodological rigour is not solely the responsibility of the research team. Once a manuscript is submitted for peer review, journal editors and reviewers play a crucial role in upholding standards by challenging or rejecting scientific manuscripts that do not meet the necessary rigour. As researchers continue to assess gender imbalances in sports-related publication volume [35, 36], it is equally essential for journals to resist the temptation to prioritise quantity over quality, maintaining a commitment to publishing high-quality, robust research.

## E—Evidence-Based Practice

Applied research should strive to be translated into practical, evidence-based applications. However, research outputs often remain limited to scientific papers, which can be inaccessible to non-academic audiences such as football players, coaches, and performance and medical staff. This raises questions about whether a research paper alone can effectively transfer knowledge in women’s football. Therefore, researchers should consider who their target audience is and evaluate the most appropriate ways to communicate their findings, including the format, terminology and accessibility. While scientific publications are essential for ensuring the research undergoes a professional and constructive independent review, it is equally important that the findings are shared with end-users in ways that are easy to access and understand. Today’s technology offers a range of tools for knowledge dissemination, including podcasts, meetings, infographics, social media content and courses (online and in-person). Researchers may benefit from incorporating these approaches during the project development phase to ensure the necessary time, funding and resources are allocated to translate findings into practice. It is also crucial to align research with the real-world needs of those who will use the evidence. Engaging stakeholders throughout the research process—from development to dissemination—can help ensure the outcomes are relevant and useful. An example of this approach is seen in the FIFA concussion knowledge transfer project [32, 33], where collaboration between researchers and practitioners facilitated the effective application of the research findings.

## N—Nurturing Talent: Focus on Long-Term Athlete Development from Youth to Elite Levels and Ensure Career Longevity

While talent development in women’s football involves many contributors—including coaches, support staff, administrators and researchers—this section focuses specifically on players. This focus reflects the important role that long-term player development plays in shaping performance, health outcomes and career longevity. Given the unique developmental pathways and transitional challenges that women’s players encounter, targeted research in this area remains a key priority to ensure that the growth of the women’s game is supported by evidence-based practice across the athlete’s lifespan.

**Fig. 3:**
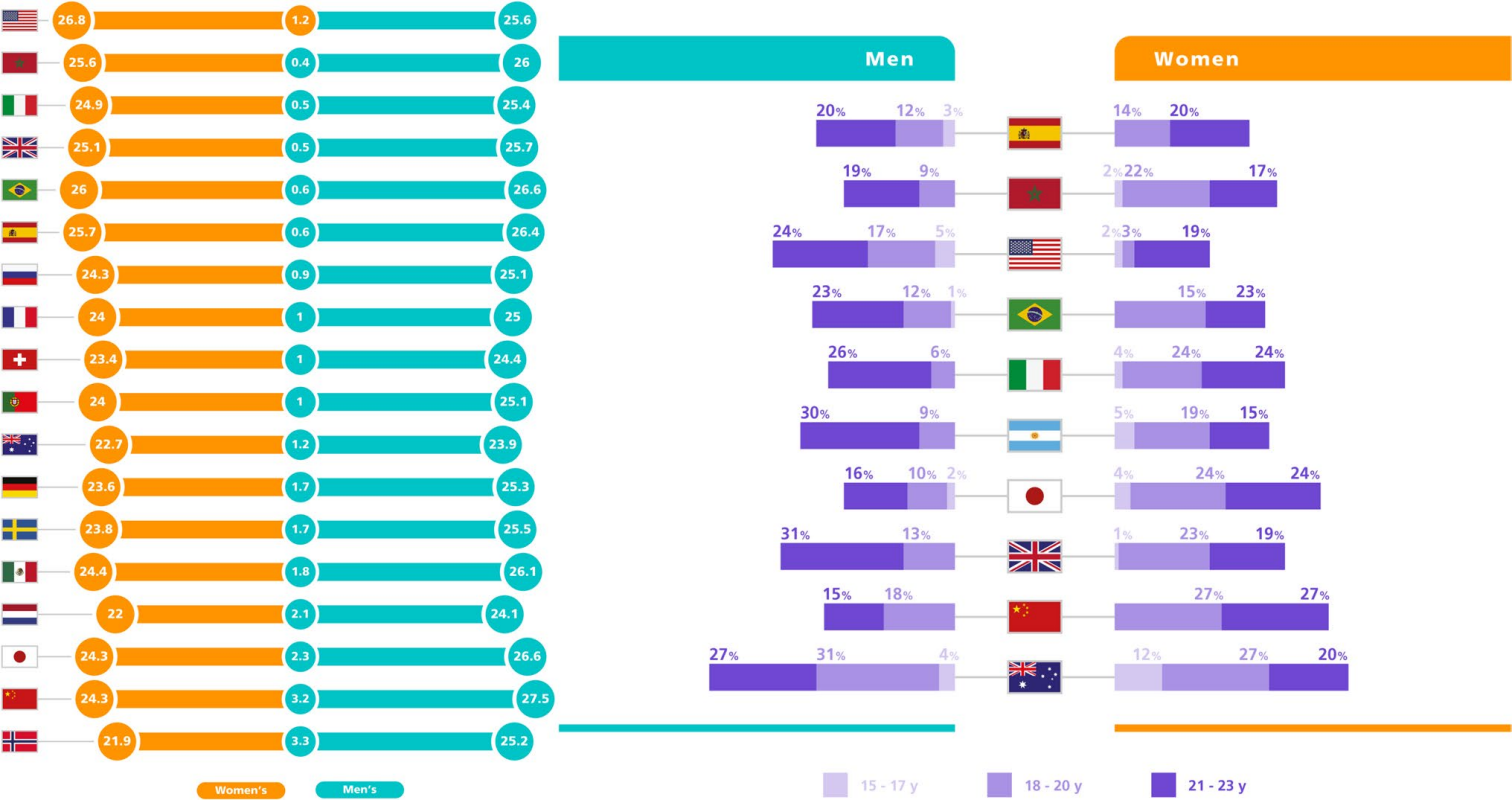
Mean age (left) and proportion (right) of players in specified age ranges across professional men’s and women’s football leagues. Data collated via Transfermarkt, Wyscout and Soccerdonna databases

**Fig. 4:**
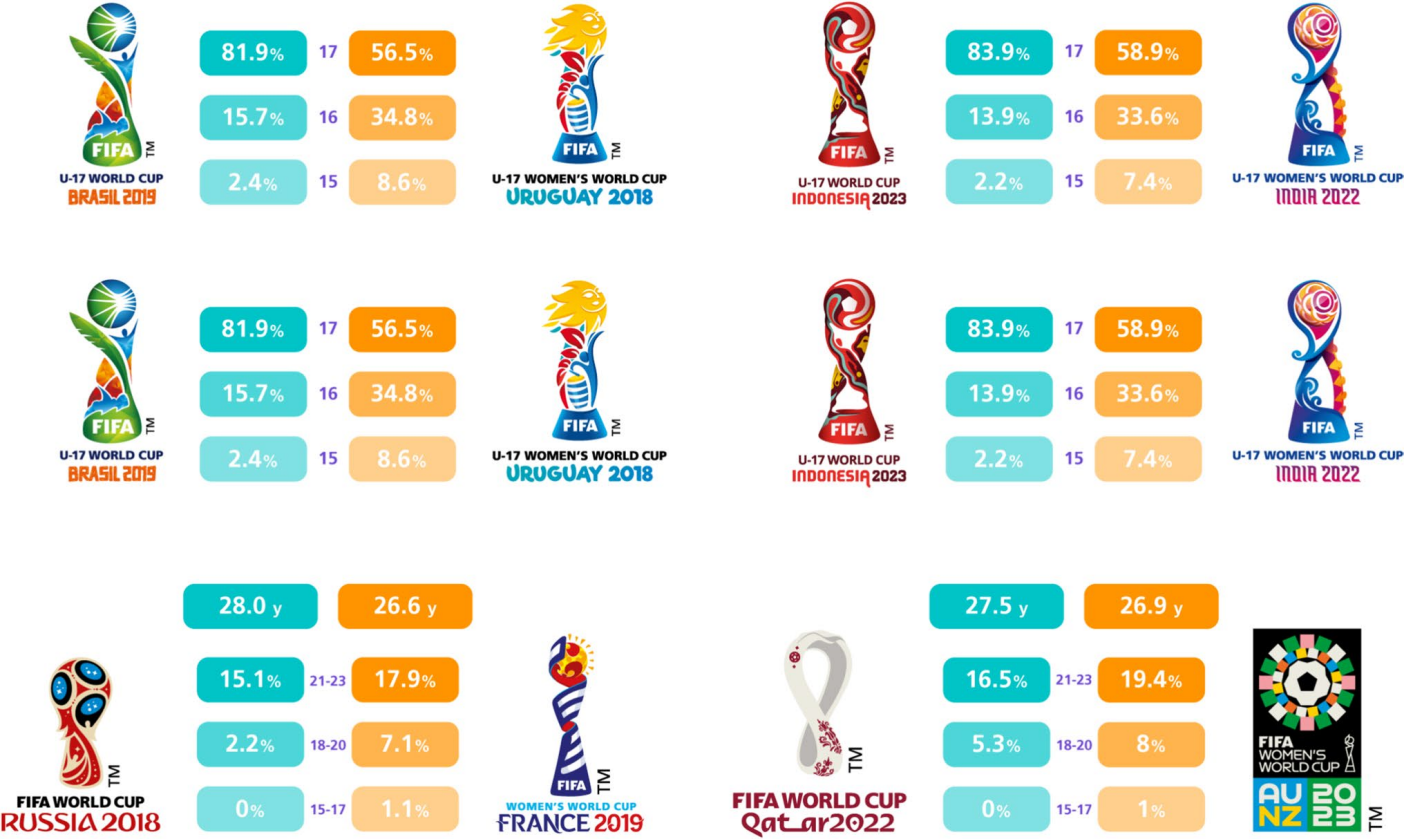
Mean age and proportion of male and female players in specified age ranges in the previous two men’s and women’s U17s (**A**), U20s (**B**) and Senior World Cups (**C**)

The temporal development and career trajectories of women footballers are unique [37, 38]. Reflecting on trends observed in professional women’s football leagues (Fig. 3), national team selections reveal a distinct pattern where younger players, particularly those under 20 years, are increasingly represented in senior squads competing at World Cup tournaments. This trend extends to youth competitions, where the U17 and U20 Women’s World Cups showcase a higher proportion of younger players (approximately three times as many 15-year-olds and twice as many 16-year-olds compared with the men’s tournaments), highlighting an accelerated developmental pathway in women’s football (Fig. 4).

Female players often manage athletic careers alongside other life events, such as pregnancy, education and employment, particularly in emerging nations where professional sports leagues are less established [39]. In addition, a lifespan perspective is needed to consider long-term health outcomes, as female players may be more susceptible to conditions such as osteoarthritis [40] due to sex-specific factors including joint morphology, hormonal influences and injury history. These factors collectively shape the need for tailored approaches in supporting the long-term development and career longevity in women’s football.

The development of athletic talent is a complex, multifaceted process that demands a nuanced understanding of the distinct trajectories from youth to elite levels. Historically, talent development research has exhibited a pronounced gender imbalance. Comprehensive reviews spanning the past two to three decades reveal that only about 10% of studies have exclusively focused on female athletes, compared with 43–60% on male athletes [41, 42]. A critical issue in the development of women players is the global disparity in training opportunities compared with their male counterparts. Data indicate boys receive approximately 57 h more training annually between ages U12 and U15, a trend that continues into later developmental stages [43]. This disparity in training opportunities is further complicated by the differing rates and patterns of technical skill development between men and women [37, 44]. To address these challenges, a comprehensive and integrated approach to studying women’s football talent development is essential. This approach would lay the foundation for tailored training and support strategies that specifically address the unique needs and challenges faced by female athletes.

A primary area requiring attention is the interplay between biological maturation and performance development in female footballers. While general patterns of growth and maturation are understood, their specific impacts on football performance remain understudied. Notably, female athletes often experience a plateau in physical performance around peak height velocity, particularly in tasks requiring rapid lower body force application, such as sprinting and jumping [45]. This phenomenon coincides with hormonal changes which is associated with increased fat mass [45], which in combination with decreases in motor competency and neuromuscular control post-puberty [46], may contribute to inefficient movement mechanics and susceptibility to injury. However, associations between growth, maturation, hormonal status and injury risk in women’s football has a low level of evidence [47]. Consequently, there is a pressing need for more robust, longitudinal research to provide a comprehensive understanding of injury patterns across development stages, and their impact upon talent development.

From a socio-cultural perspective, nurturing women’s football talent faces unique challenges that can impact their development and career trajectories. Women athletes often face inadequate support systems, with a lack of qualified coaches [43], access to medical expertise [44] and resources, which may limit technical and tactical development [45]. These socio-cultural factors impact the youth-to-senior transitions in football, highlighting the need for gender-specific organisational structures and settings in talent selection and development to account for these differences [38]. Career longevity in women’s football is significantly influenced by the need to balance dual careers, family responsibilities and the limited professional support available. Many players face challenges in managing education (36%), caring responsibilities (20%) or secondary employment (60%) alongside their football careers [48], leading to higher stress, burnout and early retirement [49]. A key factor driving retirement is the desire to start a family, with limited support for pregnancy and motherhood within the sport [50]. Given the rapid professionalisation and increased financial investment in women’s football, ongoing research is essential to maintain the relevance of existing data and studies in this evolving context. Furthermore, the transition from youth to senior levels in women’s football remains understudied. Research should examine the factors influencing successful transitions, including the impact of early specialisation, training environment continuity and the availability of role models and mentors.

By pursuing this integrated research agenda, the scientific community can contribute to the development of evidence-based, women-specific approaches to nurturing development in football. This knowledge will be instrumental in optimising performance, reducing injury rates, extending career longevity and supporting health across the lifestyle of women footballers. Moreover, it will provide a foundation for more equitable policies and practices in football, ensuring that talent and career development strategies are effective and inclusive for all athletes, regardless of gender.

## Summary

Of course, most of the considerations mentioned in this article are not unique to women’s football. However, by looking ‘back to the future’, we can better understand the historical under-representation and research gaps in women’s football, and apply lessons learned from other fields to improve future research practices. Past research has often been limited by narrow focusses and insufficient diversity in the study population, particularly in the geographical and cultural scope. Despite women’s football being under-researched for many years, we should not be satisfied with the research being conducted. Reflecting on past limitations, it is essential to prioritise the development of robust practices already available to us, establishing strong research culture where *methodological excellence* and *open science* are the norm to produce research that is easily accessible to ensure *evidence-based practice* within the *world-wide representation* of women’s football. Especially considering the growing youth participation to *nurture the talent* and, hence, longevity of ‘the beautiful game’ (Fig. 1). By combining lessons from past challenges with forward-thinking approaches such as co-design, ‘red-teams’, pre-study peer reviews, reporting guidelines and data sharing protocols, women's football research can transcend the gender-data knowledge gap, to a new standard of rigor and translational impact.

## Declarations

### EDI statement

Our author team consists of one female and one male author: representing mid-career researchers working in elite and grassroot football research, with two nationalities and living in two different countries over two continents.
